# Regulatory role of vitamin D in T-cell reactivity against myelin peptides in relapsing-remitting multiple sclerosis patients

**DOI:** 10.1186/1471-2377-12-103

**Published:** 2012-09-24

**Authors:** Laia Grau-López, Maria Luisa Granada, Dàlia Raïch-Regué, Mar Naranjo-Gómez, Francesc E Borràs-Serres, Eva Martínez-Cáceres, Cristina Ramo-Tello

**Affiliations:** 1 Department of Neurosciences, Hospital Universitari Germans Trias i Pujol, Carretera del Canyet s/n, Badalona, Barcelona, 08916, Spain; 2 Department of Biochemistry, Hospital Universitari Germans Trias i Pujol, Badalona, Barcelona, Spain; 3 Laboratory of Immunobiology for Research and Diagnostic Applications (LIRAD), Blood and Tissue Bank (BST), Institut Germans Trias i Pujol. Department of Cell Biology, Physiology and Immunology, Universitat Autònoma de Barcelona, Barcelona, Spain

**Keywords:** Multiple sclerosis, T-cell proliferation, Vitamin D

## Abstract

**Background:**

Low levels of plasma 25-hydroxyvitaminD (25(OH)D) are associated with a higher incidence of multiple sclerosis (MS) due to the immune suppressive properties of vitamin D.

The aim of this study was to determine the correlation between plasma 25(OH)D concentrations and clinical and immunological variables in a cohort of multiple sclerosis patients.

**Methods:**

Plasma 25(OH)D concentrations were evaluated in summer and winter in 15 primary progressive MS (PPMS) patients, 40 relapsing- remitting MS (RRMS) patients and 40 controls (HC). Protocol variables included demographic and clinical data, radiological findings and immunological variables (oligoclonal bands, HLADR15 and T-lymphocyte proliferation to a definite mix of 7 myelin peptides).

**Results:**

During the winter, plasma concentrations were significantly lower in RRMS patients compared to HC, whereas no differences were found in summer. No relationships were found between plasma 25(OH)D concentrations and clinical or radiological variables. RRMS patients with a positive T-cell proliferation to a mix of myelin peptides (n = 31) had lower 25(OH)D concentrations.

**Conclusions:**

25(OH)D is an immunomodulatory molecule that might have a regulatory role in T-cell proliferation to myelin peptides in RRMS patients.

## Background

Multiple sclerosis (MS) is a chronic inflammatory, neurodegenerative disease of the central nervous system of unknown aetiology [1]. It is currently considered to be a multi-causal, autoimmune disease, with genetic and environmental factors playing a role. Vitamin D deficiency has long been considered a possible causal factor of MS owing to the apparent inverse relationship between MS prevalence and sun exposure, a major factor in the production of vitamin D in humans [2,3]. Vitamin D is a modulator of calcium homeostasis; however, its role in the pathogenesis of MS is explained by its strong immune modulating potential. Vitamin D exerts a direct effect on T-lymphocyte proliferation against myelin peptides, cytokine production and cell-cycle progression [4]. Moreover, direct effects of vitamin D on dendritic cells (DC) have also been described. Vitamin D-conditioned DC are able to suppress Th1 cell proliferation and induce apoptosis [5,6].

Recent works studied serum levels of vitamin D metabolites in MS patients. Low 25(OH)D levels in healthy adolescents have been associated with a higher risk of developing MS in later life [7]. Moreover, recent observational studies established an association between vitamin D level status and clinical or immunological variables. MS patients with lower vitamin D levels have been shown to have a higher relapse risk and greater disability [8-10]. High 25(OH)D levels have also been associated with improved Treg function and with skewing of the Th1/Th2 balance towards Th2 [11]. However, clinical trials did not draw definitive conclusions regarding improvement in MS patients treated with vitamin D compounds [12]. Therefore, the relationship between plasma 25(OH)D concentrations and different clinical or immunological parameters of the disease need to be further characterised.

The aim of this study was to compare plasma 25(OH)D concentrations between MS patients and healthy controls (HC). The relationship between plasma 25(OH)D concentrations and clinical, radiological and immunological variables of MS patients was also assessed.

## Methods

### Patients

Patients of Catalonia (Spain) were clinically diagnosed with MS according to McDonald’s criteria [13]. Patients were subdivided into two groups: relapsing -remitting multiple sclerosis (RRMS) (n = 40) and primary progressive multiple sclerosis (PPMS) (n = 15); the clinical course of the disease was defined following Lublin and Reingold (1996) criteria. Recruitment was limited to men and women aged 18 to 55 years, with an Expanded Disability Status Scale (EDSS) of 0 to 5.5 [14]. Exclusion criteria included relapse, treatment with dietary supplements, immunosuppressive therapies at any time prior to enrolment and treatment with interferon beta, glatiramer acetate and corticosteroids within the previous 3 months. Forty HC with no significant demographic differences (place of residence, race/ethnicity, age and sex) compared with RRMS patients were selected. PPMS patients are usually older than RRMS and more frequently men than women. All donors gave their written informed consent and the study was approved by the Ethics Committee of Clinical Research of Hospital Germans Trias i Pujol (reference number: EO-06-030).

### Protocol variables

Protocol variables included demographic data (sex, age), neurological dysfunction (defined according to EDSS scale), number of previous relapses, annualised relapse rate, years of evolution, number of contrast-enhancing lesions and number of T2 lesions on brain magnetic resonance imaging (MRI) performed, for this study, within the three months prior to the winter months and during the winter months (from September to February). Brain MRI scans were performed in all patients with a 1.5-Tesla whole-body MRI (Philips) according to a previously-established protocol including T1-weighted spin-echo axial slices with and without the application of gadolinium, T2-weighted spin-echo axial slices and fluid-attenuated inversion recovery (FLAIR). Immunological variables included HLA-DR15 (performed by Polymerase Chain Reaction, Olerup SSP HLA low resolution), oligoclonal bands in cerebrospinal fluid (performed with the kit Helena BioSciences Europe, United Kingdom) and T-lymphocyte proliferation to a definite mix of 7 myelin immunodominant MS-related peptides (MBP13-32, MBP111-129, MBP146-170, MBP83-99, MOG1-20, MOG35-55, PLP139-154), performed during winter months, at concentrations of 10 μM of each peptide as previously described [15]. Twelve replicate wells with 10 μM of each peptide plus twelve without antigen (negative control) were prepared. After 6 days, cells were pulsed for 16 hours with 1 μCi/well ^3^[H] thymidine (Amersham, GE, Healthcare). Proliferation was determined as a function of incorporated radioactivity (cpm) and measured by scintillation counting (1450 Microbeta; Trilux Wallac, Finland). Individual wells were considered positive when cpm were more than 3-fold the standard deviation (SD) over the average cpm of the 12 negative control wells. Individuals showing ≥ 50% positive wells were classified as responders and the remainder as non-responders.

### Blood samples

Data and blood samples were collected at two time points: during winter months (December, January and February) and during summer months (June, July and August). The same patients were sampled in both seasons.

Blood samples were obtained by venepuncture. After centrifugation, plasma was aliquoted and frozen at −80°C until assayed for 25(OH)D. Plasma 25(OH)D concentrations were measured using a competitive electro-chemiluminescence immunoassay with the Roche Elecsys vitamin D3 (25-OH) on a Roche Modular E170 analyser. Inter-assay coefficient of variation (CV) was 6.7% and 5.0% at serum concentrations of 24.3 ng/ml and 39.6 ng/ml, respectively. Assay sensitivity was 4 ng/mL. The reference range for plasma 25(OH)D concentrations was 15.5 - 41.7 ng/mL

### Statistical analysis

25(OH)D concentrations were categorised as: plasma concentrations <20 ng/ml considered “insufficient” and plasma 25(OH)D concentrations ≥20 ng/ml considered “sufficient” as reported previously [16]. This cut-off for 25(OH)D was arbitrarly selected because a concentration <20 ng/mL has been accepted as deficient for the maintenance of adequate calcium metabolism [17]. However, the optimal level for other vitamin D functions remains uncertain and might be higher (>30 ng/ml or even >40 ng/mL). Plasma 25(OH)D concentrations were also analysed as a continuous variable.

Departure from normality was assessed using the Kolmogorov–Smirnov distribution test. Descriptive results are expressed as mean and standard deviation (SD) or median and interquartile range (IR).

As a continuous variable, plasma 25(OH)D concentrations were tested for differences between MS patients and HC using the Mann–Whitney *U* test. In MS patients, the association between plasma 25(OH)D concentrations and age, number of relapses, years of disease evolution, EDSS score, number of T2 and contrast-enhancing lesions was calculated using Spearmans correlation. The relationship between plasma 25(OH)D concentrations and T-lymphocyte proliferation against myelin peptides (yes/no) was measured using the Mann–Whitney *U* test.

As a dichotomous variable (sufficient/insufficient 25(OH)D), chi-square analyses or Fisher’s exact tests were applied to analyse the relationship of T lymphocyte proliferation against myelin peptides (yes/no) and the Mann–Whitney *U* test was applied to analyse the association with age, EDSS, years of evolution, number of previous relapses, annualised relapse rates, number of contrast-enhancing lesions and number of T2 lesions. The independent influence of baseline characteristics on plasma 25(OH)D concentrations was assessed by logistic regression analysis. Variables related to lower plasma 25(OH)D concentrations in the univariate analysis with a P value < 0.05 were included in the model (Enter approach).

Differences in some of the variables between seasons were analysed using paired statistical analyses such as the Wilcoxon test.

All statistical analyses were performed using the Statistical Package for Social Sciences (SPSS/Windows version 17.0; SPSS Inc. Chicago, IL, USA). P values < 0.05 were considered significant.

## Results

### Clinical characteristics of subjects

Ninety-five subjects were assessed: 40 RRMS patients, 15 PPMS patients and 40 HC. Demographic and clinical characteristics of patients and controls are summarised in Table 1. Thirty-five patients (87%) of the RRMS group were women. Mean age of RRMS patients at inclusion was 40 years (± 7.8), median EDSS 2.5 [1.5-3.5] and mean disease duration 11 years (± 6.7). Of the PPMS group, 7 patients (45%) were women. Mean age of PPMS patients at inclusion was 48.5 years (± 12.7), median EDSS 3.5 [2.0-5.5] and mean disease duration 10 years (± 5.3). Thirty patients (70%) of the HC group were women. Mean age of HC at inclusion was 38 years (± 6.8).

**Table 1:** Demographic and clinical characteristics of the study population

	**RRMS**	**PPMS**	**HC**
**N**	40	15	40
**Age (Years)** a	40 ±7.8	48.5 ±12.7	38 ±6.8
**Female (%)**	87%	45%	75%
**EDSS**	2.5 [1.5-3.5]	3.5 [2.0-5.5]	--------
**Disease duration (yrs)**	11 ±6.7	10 ±5.3	--------

a. Values are expressed as mean ± SD. *RRMS* = relapsing-remitting multiple sclerosis; *PPMS* = primary progressive multiple sclerosis; *HC* = healthy controls; *EDSS* = expanded disability status scale.

### Plasma 25(OH) D concentrations between MS patients and HC

**Figure 1:**
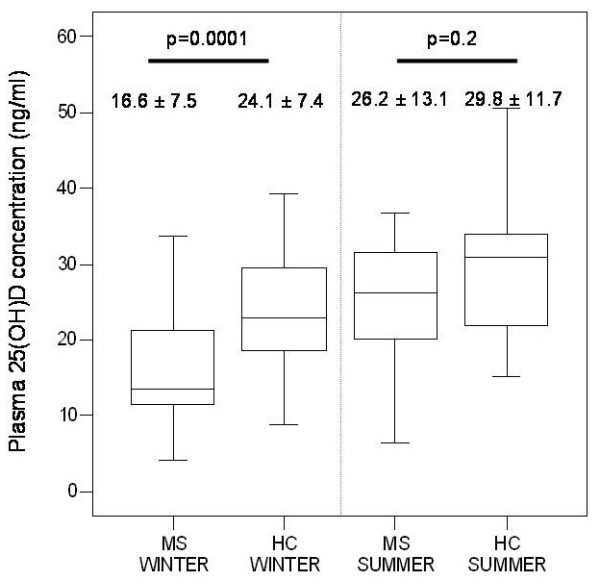
**Plasma 25(OH)D concentrations between RRMS patients and HC during summer and winter.** Plasma 25(OH)D concentrations (mean + SD) were significantly lower (p = 0.0001) in RRMS patients (n = 40) compared to HC (n = 40) during the winter months. Differences in plasma 25(OH)D concentrations were not detected (p = 0.2) between patients and HC during the summer months. RRMS and HC had higher plasma 25(OH)D concentrations in summer months compared to winter months (p = 0.05 and p = 0.03, respectively). MS: Multiple sclerosis patients. HC: Healthy controls.

During winter, plasma 25(OH)D concentrations were significantly lower in RRMS patients compared to HC (16.6 ± 7.5 ng/ml vs 24.1 ± 7.4 ng/ml, respectively, p = 0.0001). In contrast, PPMS patients showed 25(OH)D concentrations similar to controls (22.1 ± 6.1 ng/ml vs 22.8 ± 7.1 ng/ml, p = 0.7). Differences were not detected during summer months between RRMS and HC (26.2 ± 13.1 ng/ml vs 29.8 ± 11.7 ng/ml, p = 0.2) or between PPMS and HC (27.2 ± 10.3 ng/ml vs 30.5 ± 11.7 ng/ml, p = 0.6) (Figure 1).

During winter, a higher percentage of RRMS patients had insufficient plasma 25(OH)D concentrations (<20 ng/ml) compared to HC (65% vs 45%, p = 0.033). During summer the number of individuals with 25(OH)D deficiency was low (7 RRMS patients, 2 PPMS patients and 6 HC) (data not shown).

### Association between plasma 25(OH)D concentrations and demographic, clinical, radiological and immunological disease characteristics in the MS cohort

To investigate the potential pathogenic role of 25(OH)D concentrations, demographic, clinical, immunological and MRI data were compared among RRMS patients with 25(OH)D sufficiency (>20 ng/ml) versus insufficiency (<20 ng/ml) at two time points: summer and winter.

During summer, differences in demographic, clinical and immunological variables were not found among RRMS patients with 25(OH)D sufficiency (>20 ng/ml) versus insufficiency (<20 ng/ml) (data not shown). T-cell proliferation against myelin peptides was not analysed in summer months.

During winter (Table 2), no significant differences in demographic data were found between patients with insufficient and sufficient 25(OH)D. No significant association was found between plasma 25(OH)D and clinical variables such as number of previous relapses or years of evolution. However, patients with plasma 25(OH)D <20ng/mL had higher EDSS scores than patients with plasma 25(OH)D > 20 ng/mL (2.0 [1.5-4.5] vs 3 [1–5.0], p = 0.02). No significant association was found between plasma 25(OH)D concentrations and brain MRI parameters (number of contrast-enhancing lesions and number of lesions in T2), positivity for HLADR15 or presence of oligoclonal bands in cerebrospinal fluid. In contrast, 88.5% of patients with insufficient 25(OH)D concentrations (<20 ng/ml) had positive T cell proliferation to the mix of myelin peptides, and only 35.7% of patients with sufficient 25(OH)D concentrations (>20 ng/ml) had positive T-cell proliferation to the mix of myelin peptides (p = 0.001). Positive T-cell proliferation was independently associated with lower 25(OH)D concentrations (odds ratio 3.1; [95% CI, 1.29–5.15]) after adjustment for age, number of previous relapses and EDSS score.

**Table 2:** Variables associated with sufficient ((25(OH)D >20ng/ml) vs insufficient (25(OH)D) <20ng/ml) plasma concentrations in RRMS patients during winter

	**All patients n = 40**	**25(OH)D >20 ng/mL n = 16**	**25(OH)D <20 ng/mL n = 24**	**p-value**
**Age**	40 ± 7.8	40.6 ± 9.2	41.8 ± 7.05	0.5
**% Female**	35 (87%)	13 (92.9%)	22 (84.6%)	0.45
**Years of evolution**	11 ± 6.7	10.3 ± 7	12.1 ± 6.7	0.4
**Number of previous relapses**	6 ± 4.2	4.4 ±3.4	7.3 ± 4.2	0.08
**Annualised relapse rates**	0.35 ±0.2	0.4 ±0.2	0.3 ±0.2	0.2
**EDSS**	2.5 [1–3.5]	2.0[1.5-4.5]	3[1–5.0]	0.02
**Oligoclonal bands**	23 (54%)	9 (56.2%)	14 (58.3%)	0.28
**HLADR15 positive**	8 (21%)	4 (28.6%)	4 (15.4%)	0.3
**Proliferation of T cells to myelin peptides**	28 (70%)	5 (35.7%)	23 (88.5%)	0.001^*^
**Number of lesions in T2**	42.5 ± 36	30.7 ± 23.7	44 ± 38	0.4
**Number of contrast- enhancing lesions**	0.85 ± 2	0.4 ± 0.9	1.2 ± 2.6	0.8

* = statistically-significant; *RRMS* = relapsing-remitting multiple sclerosis; *25(OH)D* = 25hydroxyvitamin; *EDSS* = expanded disability status scale.

**Figure 2:**
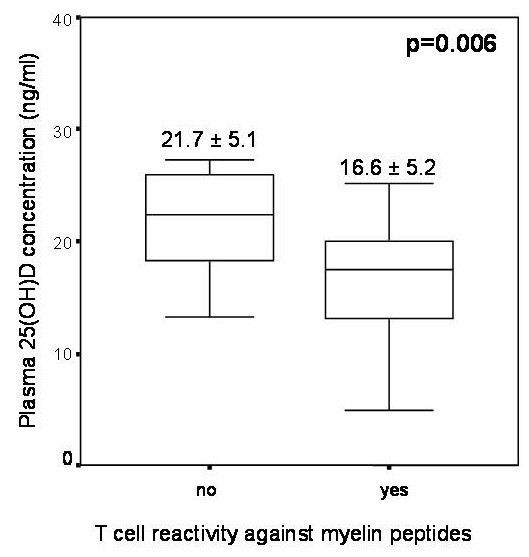
**Plasma 25(OH)D concentrations in patients with or without T-cell proliferation against myelin peptides.** RRMS patients were grouped according to negative or positive T-cell reactivity. During winter, RRMS patients with positive T-cell proliferation had lower plasma 25(OH)D concentrations (mean + SD) than patients with negative T-cell proliferation (p = 0.006).

Plasma 25(OH)D concentrations were also analysed as a continuous variable, and an association with demographic, clinical and radiological variables was not found (data not shown). Nevertheless, Figure 2 demonstrates that RRMS patients with positive T-cell proliferation (n = 31) had lower plasma 25(OH)D concentrations than patients with negative T-cell proliferation (n = 9) (16.6 ± 5.2 vs 21.7 ± 5.1, p = 0.006). HC with positive T-cell proliferation (n = 12) had similar plasma 25(OH)D concentrations to HC with negative T-cell proliferation (n = 28) (20.6 ± 2.2 vs 21.2 ± 3.2, p = 0.3).

## Discussion

The results of this study demonstrate that RRMS patients had lower plasma 25(OH)D concentrations compared with HC during the winter months. In agreement with our results, previous studies [18] demonstrated summer/winter differences in vitamin D status between MS patients and HC. In our opinion, differences in plasma 25(OH)D concentrations during summer could not be detected because sun exposure in these months led to an increase in plasma 25(OH)D concentrations in RRMS patients to levels similar to HC.

In this study, the RRMS patients selected were not on disease-modifying drugs (DMD) since we aimed to analyse plasma 25(OH)D concentrations, T-cell reactivity against myelin peptides and the association between vitamin D status and clinical, radiological and immunological variables without the interference of immunomodulatory treatment. The main reasons for patients not receiving DMD were: they did not fulfil the criteria for receiving DMD in our area (two relapses in the previous three years) or did not wish to be treated with DMD.

In this study, PPMS patients had similar plasma 25(OH)D concentrations to HC, as previously reported [4]. Consistent with these observations, a growing body of evidence indicates that distinct pathogenic processes mediate brain damage in different groups of MS patients [19].

Several efforts have been made to correlate clinical, radiological and immunological MS data with plasma 25(OH)D concentrations Reviewed in [20]. However, a relationship between vitamin D and clinical activity of the disease has not been established. In accordance with our results, a significant correlation of plasma 25(OH)D with gadolinium-enhanced or T2-weighted MRI has not been reported. In contrast, previous studies demonstrated a correlation between low 25(OH)D levels and high EDSS score or an increased risk of relapse [8-10], which concurred with our observation that patients with 25(OH)D insufficiency had higher EDSS scores than those with sufficient 25(OH)D. We believe there are two possible explanations. Low 25(OH)D levels could induce a greater neurological disability due to an aetiopathogenic factor. Disabled patients with high EDSS have loss of autonomy, gait impairment and a more sedentary lifestyle than patients with low EDSS; therefore, they are less likely to be exposed to sun, which could lead to less endogenous synthesis of 25(OH)D.

Furthermore, lower plasma 25(OH)D concentrations were observed in patients with positive T-cell proliferation against myelin peptides who had a higher number of previous relapses and greater disability than unresponsive patients. A limitation of this study is that our cohort was too small to permit a definitive assessment of the clinical outcome measures. However, we believe increasing 25(OH)D levels exert a possible beneficial effect on improving not only immunological variables such as T-cell proliferation against myelin peptides but also the clinical parameters. In this context, previous studies [12] reported that MS patients treated with vitamin D supplements had a reduced number of relapse events and a persistent reduction in T-cell proliferation compared to controls.

To define the role of vitamin D as an immunomodulatory agent, the relationship between plasma 25(OH)D concentrations and immunological parameters such as proliferation of T-cell reactivity against myelin peptides was analysed in this study. Previously, we demonstrated that T-cell proliferation against a selected mix of 7 myelin peptides was more common in RRMS patients than in HC, which supports their pathogenic significance in MS [21]. Interestingly, patients with positive T-cell proliferation against myelin peptides had low levels of plasma 25(OH)D. Correale et al. [4] demonstrated by *in vitro* coculture with 1,25(OH)2D3 that the proliferation of myelin basic protein (MBP)-specific T cells was significantly inhibited. These results suggest that vitamin D may play an immunomodulatory role in T-cell proliferation to myelin peptides. Moreover, previous studies reported an inhibitory effect of vitamin D on Th1 cell function and a beneficial effect on Th2 and Treg cells *in vitro *[9]. Furthermore, 1,25-(OH)2D proved to exert a beneficial effect on clinical and histological disease features in an experimental allergic encephalomyelitis animal model of MS [22].

Vitamin D acts through the vitamin D receptor (VDR). Certain polymorphisms of the VDR may modify vitamin D function. Some studies showed that polymorphisms are significantly more common in MS [23]. Nevertheless, other studies found no differences in VDR gene polymorphisms between MS and control groups [24]. A limitation of this study was that we did not determine the polymorphisms of the VDR, which should therefore be assessed in further studies on vitamin D and MS.

## Conclusions

Vitamin D is an immune modulator; however its clinical effect in MS remains unclear. This study demonstrated that MS patients have lower plasma 25(OH)D concentrations during wintertime compared to HC. Plasma 25(OH)D concentrations were also associated with T-cell reactivity against myelin peptides. These observations provide support for further research in this direction to confirm the potential benefit of vitamin D supplements in MS patients.

## Competing interests

The authors declare that they have no competing interests.

## Authors’ contributions

LGL participated in the design of the study, performed most of the experiments, participated in the statistical analysis and interpretation of data and drafted the manuscript. MLG carried out the determination of plasma 25-hydroxyvitaminD and helped in the statistical analysis and interpretation of data and helped in writing the manuscript. DRR contributed in culture techniques of T-lymphocyte proliferation to myelin peptides and revised the manuscript. MNG participated in culture techniques, interpretation of data and revised the manuscript. FBS participated in the design of the study and revised the manuscript. EMC conceived and designed the study and helped to draft manuscript. CRT participated in the design of the study, supervised the research and revised the manuscript. All authors read and approved the final manuscript.

## Pre-publication history

The pre-publication history for this paper can be accessed here:

http://www.biomedcentral.com/1471-2377/12/103/prepub
